# Discoblock-Associated Subarachnoid Hemorrhage: A Case Report

**DOI:** 10.1155/crnm/5167713

**Published:** 2025-10-28

**Authors:** Ahmed M. Sonbol, Abdel Fattah Ewais, Farid Kassab, Khalid Mohammed Ali Khalid, Mohammed Awad A. Mohammed, Hassan Sirajaldeen Alhassan Ali, Mohammed M. Elgack

**Affiliations:** ^1^Department of Orthopedics, Dr. Soliman Fakeeh Hospital, Jeddah, Saudi Arabia; ^2^Department of Medical Education, King Abdulaziz University, Jeddah, Saudi Arabia; ^3^Orthopedic Department, International Medical Center, Jeddah, Saudi Arabia; ^4^Andalusia Hospital, Jeddah, Saudi Arabia; ^5^Neurosurgery Department, International Medical Center, Jeddah, Saudi Arabia; ^6^King's College London Hospital, Jeddah, Saudi Arabia

**Keywords:** cefazolin, discoblock, discography, iohexol, subarachnoid hemorrhage

## Abstract

The discoblock procedure entails the administration of an anesthetic agent during discography to pinpoint the origin of spinal pain in challenging diagnostic scenarios. Known for its minimal complication rate, the most frequently documented adverse effect is discitis. This case report introduces a novel observation of subarachnoid hemorrhage following a discoblock procedure. Initially presenting with persistent lower back pain, the patient exhibited dehydrated lumbar discs on imaging, characterized by altered T2 signal intensity and a diffuse disc bulge impacting the anterior thecal sac at the L4-5 level, alongside degenerative scoliosis at L2-3. These findings suggested the potential origins of symptoms at L4-5 or L2-3, leading to the decision to proceed with L4-5 discoblock. Symptom alleviation postdiscoblock, coupled with prophylactic cefazolin administration, indicated the necessity for further management at the L4-5 disc level. Subsequently, the patient presented with status epilepticus 9 hours later, with brain magnetic resonance imaging revealing anomalous hyperintensities in the left temporoparietal sulci and the left ambient cistern, prompting suspicion of subarachnoid hemorrhage. This study elucidates the procedural indications for discoblock, explores potential factors contributing to complications, and delves into the safety considerations surrounding this intervention.

## 1. Introduction

Discography, or discogram, was first described by Hirsch [1] and Lindblom [2] as a tool for diagnosing herniated nucleus pulposus. The development of less invasive and more accurate imaging modalities, such as magnetic resonance imaging (MRI) and computed tomography (CT), has limited the role of discography. Nevertheless, discography can be an additional diagnostic option in specific cases [3]. Discoblock is a modification of discography in which the disc being evaluated is injected with an anesthetic agent and a contrast agent to eliminate the patient's pain from the technique [4, 5]. The complication rate of lumbar discography ranges from 0% to 2.7% [6, 7]. Reported complications include discitis, epidural abscess, worsening of the low back pain, headache, acute disc herniation, pulmonary embolism, and intraprocedural nausea and convulsions [6–8].

Herein, we describe what, to best of our knowledge, is the first documented case of subarachnoid hemorrhage (SAH) as a complication of discoblock.

## 2. Case Presentation

A 52-year-old man was presented to a spinal surgery clinic in December 2011 with severe lower back pain. He had difficulty walking and could not sit for even 5 minutes. He had not responded adequately to conservative treatment including analgesia, nonsteroidal anti-inflammatory medications, physical therapy, and epidural injections.

He had undergone lateral recess decompression and discectomy at L4-5 2 years ago, with a postoperative complication of a 4 × 10 cm meningocele that had been treated conservatively. The decompression and discectomy had relieved his leg pain, but his back pain gradually increased. The patient had no other significant medical history.

**Figure 1:**
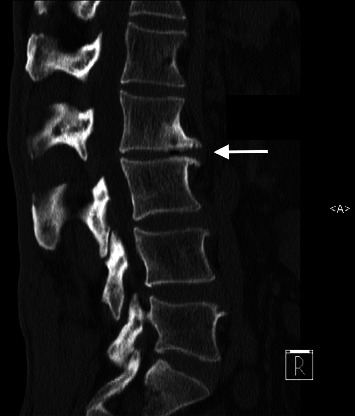
Computed tomography image of the lumbar spine shows disc space degeneration at multiple levels, predominantly at L2-3 (arrow).

**Figure 2:**
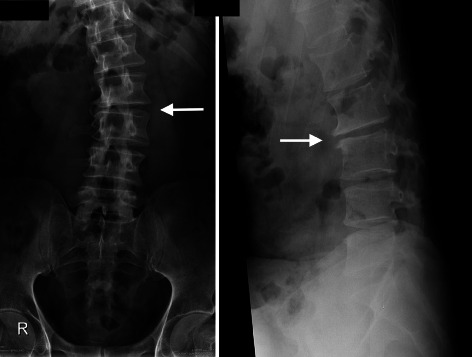
Lumbar spine radiographs show degenerative scoliosis with the apex at L2-3 (arrows).

**Figure 3:**
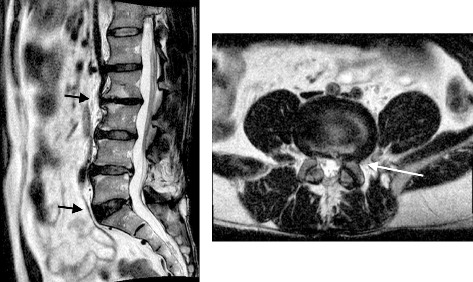
Lumbar spine magnetic resonance images show dehydrated lumbar discs with a loss of normal T2 signal intensity (black arrows). A diffuse disc bulge is seen indenting the anterior aspect of the thecal sac at L4-5 (white arrow).

Neurological examination results were normal. The patient's clinical picture was suggestive of discogenic back pain. Diagnostic imaging was requested, which revealed disc degeneration at multiple levels, most significantly at L2-3 (Figure 1), and a lumbar curvature with an apex at L2-3 (Figure 2). Furthermore, postdiscectomy changes of L4-5 degeneration, diffuse disc bulge, and bilateral recess stenosis were noted (Figure 3).

The source of patient's symptoms was thought to be either the postdiscectomy changes at L4-5 or the advanced degeneration at L2-3. We planned a diagnostic discoblock at L4-5 to confirm the source of pain depending on whether the symptoms resolved.

**Figure 4:**
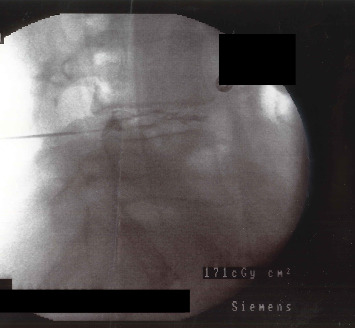
Intraoperative fluoroscopy image shows discography being performed at L4-5 with no leakage of the contrast agent.

On the next day, the patient was admitted to the operating room to undergo discography and discoblock in the prone position under conscious sedation using fentanyl, ketamine, propofol, and midazolam. Under fluoroscopy guidance, a needle was inserted at L4-5. Using the double needle technique, 2 mL of 2.5% bupivacaine hydrochloride mixed with 1 mL each of cefazolin and iohexol was injected. The needle position was fluoroscopically confirmed, and no leakage was detected (Figure 4).

The patient's condition was stable on awakening following the procedure. He reported symptom resolution and could sit comfortably with no pain. This confirmed that the pain originated from L4-5. Therefore, L4-5 interbody fusion with instrumentation was planned for a subsequent date. The patient was kept in the postanesthesia care unit for few hours, following which he was discharged. During discharge, his condition was stable and he could walk without difficulty.

**Figure 5:**
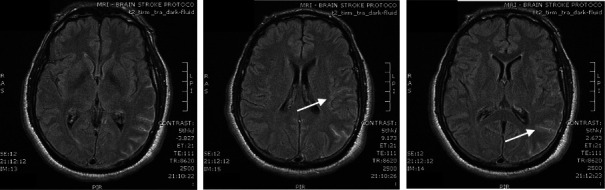
Brain magnetic resonance images show abnormal hyperintensity at the sulci in the left temporoparietal region and at the left side of the ambient cistern (arrows), raising the suspicion of subarachnoid hemorrhage.

**Figure 6:**
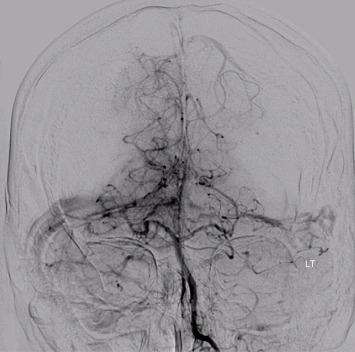
Cerebral angiogram shows no notable arteriovenous malformation or aneurysm.

**Figure 7:**
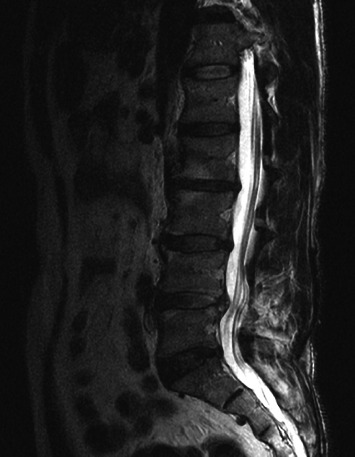
Lumbar spine magnetic resonance image shows unremarkable changes compared with the prediscography image findings.

Nine hours after the procedure, while at home, the patient began having a severe headache. Subsequently, he experienced agitation, which progressed to confusion, and convulsions. He was brought to the emergency department. There, he had another generalized tonic-clonic seizure, which progressed to status epilepticus. Therefore, treatment with intravenous antiepileptic agents was started. The patient was electively intubated and admitted to the intensive care unit for further management. Brain MRI (Figure 5) revealed SAH predominantly involving the left temporoparietal region as confirmed with CT. Conventional cerebral angiography (Figure 6) did not reveal any vascular malformation or clear source of bleeding. Lumbar puncture and cerebrospinal fluid culture was negative, excluding infectious etiology. Therefore, the patient was treated conservatively. Repeat brain CT performed after 10 days revealed gradual resolution of the SAH without hydrocephalous or increased intracranial pressure. Lumbar spine MRI (Figure 7) findings were unremarkable. The patient was discharged after 13 days. At discharge, he had headache and persistent low back pain and was receiving anticonvulsant medications.

One month later, the patient underwent L4-5 decompression, spinal fusion with instrumentation, and transforaminal interbody fusion with no complications.

## 3. Discussion

Here, we present the first reported case of SAH after discography, to the best of our knowledge. Our patient developed epilepsy secondary to SAH on the day he underwent diagnostic discoblock. No clear source of bleeding could be identified.

Complications rarely occur following discography. The most common complication is bacterial discitis. Other reported complications include cerebrospinal fluid leakage, retroperitoneal bleeding, and chronic pain [7, 9, 10]. Cefazolin premixed with a contrast agent is reportedly used to reduce the risk of discitis [11, 12]. However, cefazolin has been found to be a potent epileptogenic if injected intrathecally [13–15]. In our case, intraoperative fluoroscopy findings confirmed that cefazolin was not injected intrathecally. However, given our patient's history of postoperative meningocele, this might have a correlation. Therefore, caution must be taken before administering intrathecal antibiotics to patients undergoing discography with a history of dural tear [16, 17].

Iohexol injection (Omnipaque) is a nonionic, low-osmolar iodinated contrast agent commonly used in neuroimaging such as discography, myelography, and CT angiography. The association between iohexol and SAH is not well established in humans in the literature [18]. Although, experimental models have shown that idionated contrast media can influence the pattern and frequency of hemorrhagic transformation after reperfusion injury. But these findings pertain to parenchymal hemorrhage and do not directly implicate iohexolin the pathogenesis of SAH [19].

Unlike chymopapain, iohexol is not associated with hemorrhagic central nervous system complications in humans [20, 21]. Chymopapain and iohexol injection are both used in diagnostic and therapeutic procedures for spinal conditions, but their mechanisms of action and purposes differ. In an animal study, iohexol injection use during lumbar myelography was associated with intracranial SAH [22]. This might explain why SAH occurred in our patient.

Although various postdiscography adverse events have been reported, no cases of discography associated SAH have been reported till date. Intracranial hemorrhage following lumbar spine surgery was reported by Khalatbari et al. [23]. In such cases, the patient may be asymptomatic or experience an intense headache in the first week after surgery. Although rare, intracranial hemorrhage can occur postoperatively, with symptoms such as dural tear, cerebrospinal fluid leakage, focal neurologic symptoms, and headache [24, 25]. Our patient's first symptom was headache, which progressed to agitation, confusion, and seizures. The occurrence of a headache after discography might indicate the need for immediate brain imaging to rule out SAH [26].

Although this is the first time such a case has been reported, spontaneous SAH is a possible complication of discography and discoblock. The exact relationship remains unclear and could be an area for future research. The status epilepticus and SAH in our patient could be related to the use of cefazolin or iohexol injection, especially given the patient's history of postoperative meningocele prior to the discography procedure.

## Data Availability

The data that support the findings of this study are available from the corresponding author upon reasonable request.
